# Electroceutical Approach for Impairing the Motility of Pathogenic Bacterium Using a Microfluidic Platform

**DOI:** 10.3390/mi8070207

**Published:** 2017-06-29

**Authors:** Ryan Berthelot, Kristina Doxsee, Suresh Neethirajan

**Affiliations:** BioNano Laboratory, School of Engineering, University of Guelph, Guelph, ON N1G 2W1, Canada; rberthel@uoguelph.ca (R.B.); kdoxsee@uoguelph.ca (K.D.)

**Keywords:** electrotaxis, *P*. *aeruginosa*, cell motility, galvanotaxis, microfluidics, wound healing

## Abstract

Electrotaxis, or galvanotaxis, refers to the migration pattern of cells induced in response to electrical potential. Electrotaxis has not been explored in detail in bacterial cells; information regarding the impact of current on pathogenic bacteria is severely lacking. Using microfluidic platforms and optical microscopy, we designed a series of single- and multi-cue experiments to assess the impact of varying electrical currents and acetic acid concentrations on bacterial motility dynamics in pathogenic multi-drug resistant (MDR) strains of *Pseudomonas aeruginosa* and *Escherichia coli*. The use of the microfluidic platform allows for single-cue experiments where electrical current is supplied at a range that is biocidal to bacteria and multi-cue experiments where acetic acid is combined with current to enhance disinfection. These strategies may offer substantial therapeutic benefits, specifically for the treatment of biofilm infections, such as those found in the wound environment. Our results showed that an application of current in combination with acetic acid has profound inhibitory effects on MDR strains of *P. aeruginosa* and *E. coli*, even with brief applications. Specifically, *E. coli* motility dynamics and cell survival were significantly impaired starting at a concentration of 0.125 mA of direct current (DC) and 0.31% acetic acid, while *P. aeruginosa* was impaired at 0.70 mA and 0.31% acetic acid. As these strains are relevant wound pathogens, it is likely that this strategy would be effective against similar strains in vivo and could represent a new approach to hasten wound healing.

## 1. Introduction

Antibiotic resistance is a long-standing issue, and continues to be a growing problem around the globe. The number and prevalence of multi-drug resistant (MDR) bacterial strains is steadily increasing, and infecting more than 2 million people each year in the United States and causing some 23,000 deaths [1]. An important contributor to antibiotic resistant infection is biofilm infection; by definition, biofilms are inherently resistant to antimicrobials, warranting up to 1000 times the dose required to clear non-biofilm infections [2]. Therefore, alternative strategies to disinfection (e.g., electrocidal, electroceutical, and mechanical approaches) have gained interest and represent potentially powerful weapons against recalcitrant biofilm infection. Although the impact of current on mammalian cells is fairly well understood, information regarding its effect on pathogenic MDR bacterial strains is lacking.

A number of studies have investigated bacterial responses to electrical fields and applied current. For example, the motility of *Escherichia coli* cells has been evaluated in capillaries as a facet of soil remediation initiatives [3], and *Tetrahymena pyriformis’* electrotactic behaviors have been investigated with the goal of developing a microrobot [4]. The efficacy of electrical stimulation as a means of bacteriostatic control in the areas of wound healing [5,6] and biofilm remediation [7,8,9] has also been investigated in recent years. However, data on the cellular motility and electrotactic and galvanotactic behaviors of MDR bacterial species (in response to the external application of current), especially at the single cellular level, are rather limited. Electrical stimulation, particularly in the 0.050–0.500 mA direct current (DC) range, has been shown to reduce the number of viable cells, as determined by colony forming units in our previous studies. However, the underlying mechanisms by which electrical currents exert these effects in bacterial cells are poorly understood. Not only can current affect viability, but it can also impact the motility of bacterial organisms. However, the effect of current on the motility of pathogenic bacteria, especially at the single cell level, has not been extensively explored.

The migration and motility of cells plays an important role in wound healing. Diverse cellular factors, such as chemical and electrical cues, guide the directional migration of several organisms [10]. For example, leukocytes (e.g., lymphocytes and neutrophils) can detect and follow gradients of tissue-derived chemoattractants [11] and electric fields generated endogenously at wound sites to foster healing and antimicrobial defense [12]. Therefore, the strategic application of current could augment natural wound healing responses in mammalian cells, while targeting pathogenic bacteria. MDR strains of *Pseudomonas aeruginosa* and *Escherichia coli* are of particular interest in the wound environment, as they are common wound pathogens that possess motility systems critical to biofilm formation; directed motility toward a surface and the eventual loss of the flagella are essential steps in biofilm formation in these organisms [13]. *Pseudomonas aeruginosa*, a major cause of nosocomial infections, frequently infects open wounds and can cause sepsis and necrosis [14]. This organism infects approximately one-third of all burn wounds [15]. Additionally, *Pseudomonas aeruginosa* can have negative influences in both the industrial and environmental fields, causing dairy spoilage [16] and issues in water treatment systems [17]. Therefore, applying electric potential could be a complementary approach in addition to using chemical-based drugs to overcome the issue of recalcitrant, multi-drug resistant bacteria. Novel electroceutical technologies incorporating low voltage/current within a wound dressing have shown some promise, and could be implemented as a wearable electrical-based treatment system [18]. Therefore, understanding electrotaxis behaviors in MDR strains of bacteria will provide researchers with relevant information concerning the ranges of electric potential that could be applied to chronic or acute wound infections, depending on the infecting strains and their relative sensitivities.

A systematic understanding of the kinetics of the bacteria and the interplay mechanisms between single cells to the varying electrical properties will aid in developing enhanced electroceutical approaches that may prevent bacterial attachment and inhibit pathogenic bacterial growth in a wound microenvironment. Our study is a preliminary step towards the development of wound healing strategies through a better understanding of the relationship between single cell motility and electrical potential. Further studies using microfluidic platforms that would mimic the wound environment to validate the findings from the preliminary experiments would be essential.

Such electroceutical strategies combine chemical disinfection with the application of current. Acetic acid (AA) is one promising chemical candidate that may be effective in such strategies, as it is an effective, safe, and economical antimicrobial agent capable of inhibiting pathogenic and MDR strains of bacteria, even when these strains grow as a biofilm [15,19]. Although studies support the use of AA in combination with electrical stimulation as a disinfection strategy, additional measurements of the chemotactic and electrotactic behaviors of MDR strains are needed to engineer and fabricate an effective wound healing device. Therefore, we chose to compare the effects of AA or electrical stimulation (ES) alone (single cue) with those of a combination of AA and ES (multi-cue) on the chemotaxis of pathogenic species relevant to wound infection.

Our results show that combinations of AA and electrical stimulation are highly effective in reducing the motility of MDR strains of *P. aeruginosa* and *E. coli*, which is likely to impede infection and biofilm formation. Our results help to deepen our understanding of the effects of electroceutical approaches on pathogenic bacteria, suggesting that motility is one heavily impacted factor. This information may aid in the design of highly effective wound healing devices and/or strategies.

## 2. Materials and Methods

### 2.1. Experimental Parameter Design

Care was taken in the selection of various experimental parameters, which will be discussed herein. Our preliminary studies support that both *E. coli* and *P. aeruginosa* show little to no response to alternating current (AC) stimulation, but a bacteriostatic effect is observed when exposed to anodal and cathodal direct current (DC) [20,21]. The delivery of 0.200 mA for 4 h/day over four days reduced *P. aeruginosa* biofilms on Teflon and titanium discs [9], and exposure to 0.100 mA resulted in observable biofilm reductions after four days [14]. Further, in vivo studies have shown that skin ulcers colonized with *P*. *aeruginosa* and treated with µA cathodal DC resulted in pathogen-free ulcers within days of treatment [12]. Additional studies revealed that the delivery of current through carbon-filled electrodes to micro-organisms in intact human skin at 0.075 and 0.100 mA resulted in bactericidal effects at 4 and 24 h, beneath the positive electrode [22]. We selected *P. aeruginosa* strain BK-76, isolated from canine ear skin infections, and *E. coli* strain ATCC 8099, because these are relevant wound pathogens that exhibit antimicrobial resistance.

To minimize the risk of pH or temperature fluctuations, while selecting an effective current range, we elected to administer 0.07, 0.125, or 0.175 mA DC to the cells, as these doses in the range of 0.075–0.200 mA have bactericidal effects. Based on a study performed at the Mayo Clinic, the deployment of low dose electric current in the urinary tract was determined to be safe; a study of electrified catheters in sheep resulted in no chemical or physical changes/trauma to tissues or urine within the urinary tract when administering 0.400 mA of current [14]. However, it is unknown as to how inflamed, and possibly necrotized, skin wounds would respond to currents as great as 0.400 mA of DC, which is why we selected a lower 0.175 mA dose as the upper limit. The bacterial suspension consisted of either *P. aeruginosa* or *E. coli* suspended in filtered deionized water. This was done to reduce the complexity of the electrochemical products produced at the anode/cathode, allowing for a clearer observation of the bacterial response to current in terms of their chemotactic behavior. When the bacteria were observed microscopically in the prepared solution, they exhibited a high level of free motility.

Previous studies have shown a minimum inhibitory concentration of 0.31% AA to be an effective treatment against a multitude of tested pathogens, including MDR strains of *E. coli* and *P. aeruginosa* [15]. This same concentration of AA was also found to be effective in inhibiting biofilm formation [15]. Therefore, we chose to use 0.31% as the chemical component of our electroceutical approach (multi-cue experiments).

Electrotaxis and chemotaxis have been studied separately in mammalian cells due to the complications in designing simultaneous chemical mixing and electrical field gradients in a single device [11]. The development of specialized microfluidic devices has allowed researchers to test the impact of electric fields as well as controlled chemical gradients, although this is still challenging for practical application in a wound dressing. Therefore, we elected to use a single uniform concentration of AA in combination with the application of various currents.

### 2.2. Cell Culture

Bacterial suspensions were prepared by centrifuging cultures grown in 5 mL of Tryptic Soy Broth medium in the shaker at 200 rpm for 5 h at 37 °C. The media was extracted and centrifuged at 3750 rpm for 5 min to concentrate the cells. Upon pouring off the supernatant and redistributing the cells in filtered deionized water, the process was repeated two times. The cells were subsequently diluted with filtered deionized water and injected into the microfluidic device for viewing. The above procedure was also followed for the culturing of the cells for the electroceutical experiments, except 6.9 µL of 45% AA was added to 993.1 µL of the bacterial solution to achieve a 0.31% AA concentration prior to injection into the microfluidic channel.

### 2.3. Metrics Acquisition

**Figure 1:**
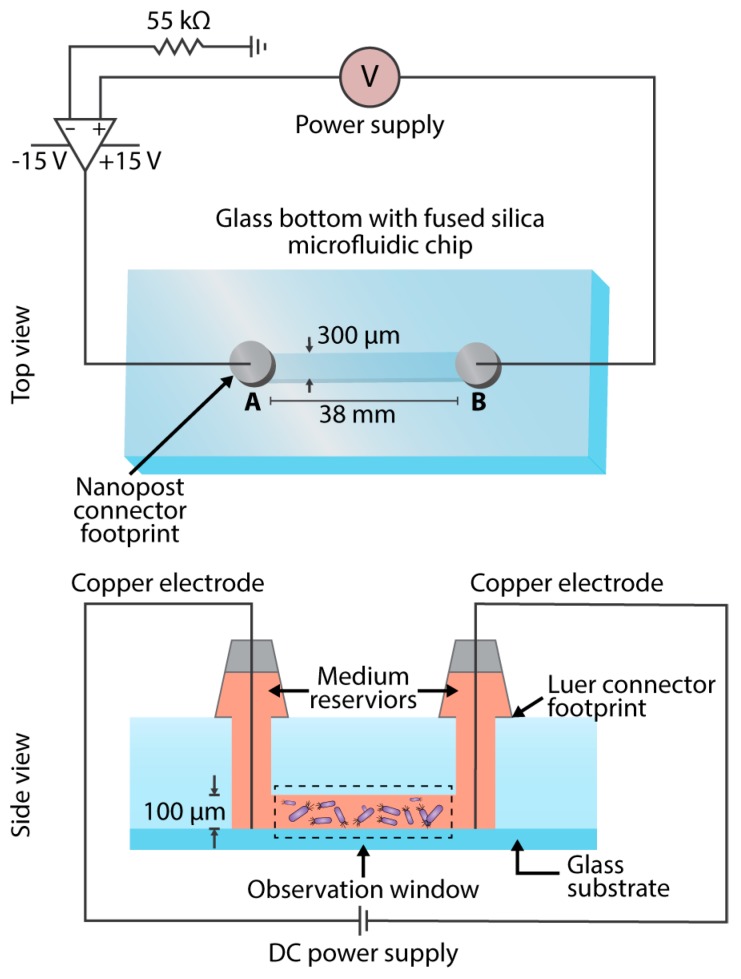
Illustration of the microfluidic experimental setup for bacterial electrotaxis assays. Schematic of the circuit used for generating the desired electric current in the investigation of the swimming dynamics of individual bacterial cells. Abbreviation: DC, direct current.

Copper electrodes (diameter, 4 mm) were inserted into ports A and B and into the bacterial suspension of the glass bottom fused silica microfluidic system (Figure 1). The desired ampere levels of 0, 0.07, 0.125, and 0.175 mA were achieved by the use of a current amplifier and a 55 Ω resistor, giving the corresponding voltage values of 0, 3.850, 6.875, and 9.625 V respectively. A Nikon Ti-U Eclipse Microscope (Nikon Instruments Inc., Melville, NY, USA) was used to image the cells at the following settings: Phase 2 contrast with the use of D & GIF filters, 40X Objective with the collar ring set to 1.3 mm, 1.5X magnification, 1.0 Gain, and recorded at 90 fps by use of the Nikon NIS-Elements software for real time imaging. The regions along the entire microfluidic channel were imaged and recorded for 40 s. The use of a flow-free microfluidic device, composed of a fused silica chip, minimizes flow-induced shear stress on cell migration and movement in a static gradient environment [23,24,25,26], and allows quantitative evaluations of cell migration in spatiotemporally complex chemoattractant fields that mimic in vivo situations [27]. In addition, the miniaturization drastically reduces the Joule heating effect [28], thereby reducing the chance of any thermotaxis by the cells. The methodology for the data analysis conducted in this study was adopted from our previous study, Wright et al. 2014 [29]. The cellular characteristic analyzed in this study includes the Forward Migration Index (FMI), where FMI X and FMI Y indicate the efficiency of the forward migration of cells and how they relate to the direction of both axes. Directness is a quantitative representation of linearity with respect to cell trajectory.

### 2.4. Statistical Tracking and Data Analysis

ImageJ (http://rsb.info.nih.gov/ij/) was used to track the cellular frame by frame coordinates, and the Ibidi Chemotaxis and Migration Tool software (Ibidi Software, Munich, Germany) was used to calculate the cellular motility metrics of the combined cell tracks. The total number of cell tracks for each setting was 70. At least five independent experiments were carried out. All of the quantitative data were presented as the mean value ± standard deviation. The Student’s *t*-test was applied to compare two distinct groups. *p* value <0.05 was considered to be statistically significant.

## 3. Results and Discussion

**Figure 2:**
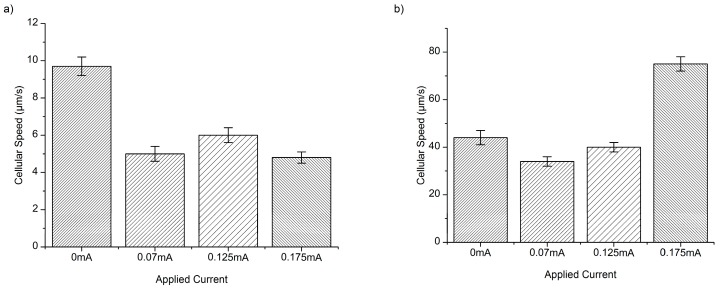
*Escherichia coli* (**a**) and *Pseudomonas aeruginosa* (**b**) cellular speed distribution in µm/s in response to electrical stimulation alone at applied current settings listed in mA. *E. coli* shows the greatest response to electrical stimulation alone at 0.07 and 0.175 mA, while *P*. *aeruginosa* shows the greatest response at 0.07 mA within the standard error.

**Figure 3:**
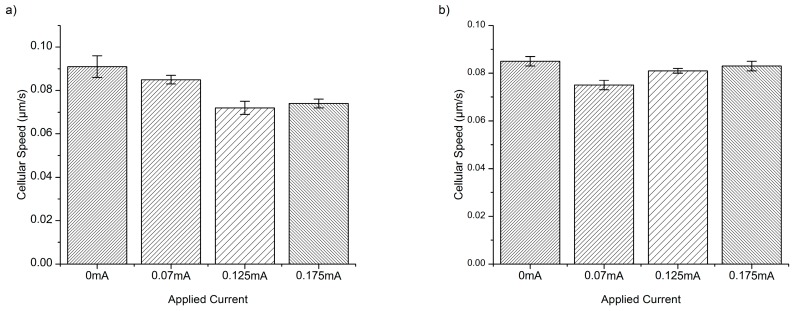
*E. coli* cellular speed distribution in µm/s in response to electrical stimulation and 0.31% acetic acid (AA) specific currents (**a**). Results indicate a great reduction in cellular speed upon electroceutical treatment. *E. coli* was equally responsive to both 0.125 mA and 0.175 mA settings. Results indicate a great reduction in cellular speed with electroceutical treatment of *P*. *aeruginosa* (**b**) at the 0.07 mA setting.

**Figure 4:**
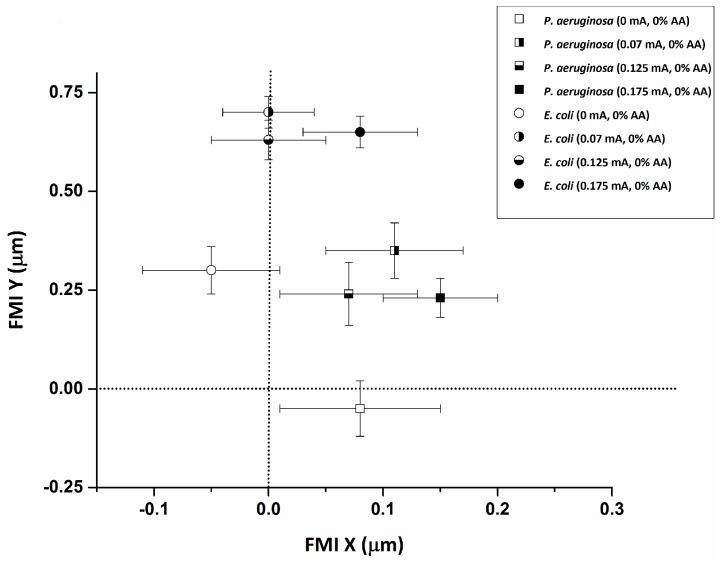
The average forward migration index (FMI) in the *x* and *y* directions for *E. coli* and *P*. *aeruginosa* in response to the application of current is plotted. The results denote increased migration along the electrotactic gradient (*y* axis) with the application of current (relative to 0 mA baseline).

Both *E. coli* and *P*. *aeruginosa* experience a reduction in cellular speed (Figure 2 and Figure 3) and an increase in the forward migration index (FMI) in response to the application of DC, along the chemotactic gradient plotted on the *y*-axis (Figure 4). There is also a significant increase in directness with current application (Table 1), relative to the baseline (0 mA). The resulting average cellular speeds for *E. coli* were 9.7 ± 0.5 µm/s, 5.0 ± 0.4 µm/s, 6.0 ± 0.4 µm/s, and 4.6 ± 0.3 µm/s for 0 mA, 0.07 mA, 0.125 mA, and 0.175 mA DC, respectively (Figure 2). The resulting average cellular speeds for *P*. *aeruginosa* were 44 ± 3 µm/s, 34 ± 2 µm/s, 40 ± 2 µm/s, and 75 ± 3 µm/s for 0 mA, 0.07 mA, 0.125 mA, and 0.175 mA DC, respectively (Figure 2). The differences in the response to current between *E. coli* and *P*. *aeruginosa*, as measured by the change in cellular speed, likely reflect differences in their motility and chemo/electrotactic sensing systems [30]. This may help to explain why there is an increase in cellular speed in *P*. *aeruginosa* at 0.175 mA (relative to baseline), while the application of any current reduces cellular speed in *E. coli*. However, 0.07 and 0.125 mA currents reduced the motility of both organisms, indicating that there may be an optimal range of current that will predictably and consistently impact several pathogenic bacterial species.

**Figure 5:**
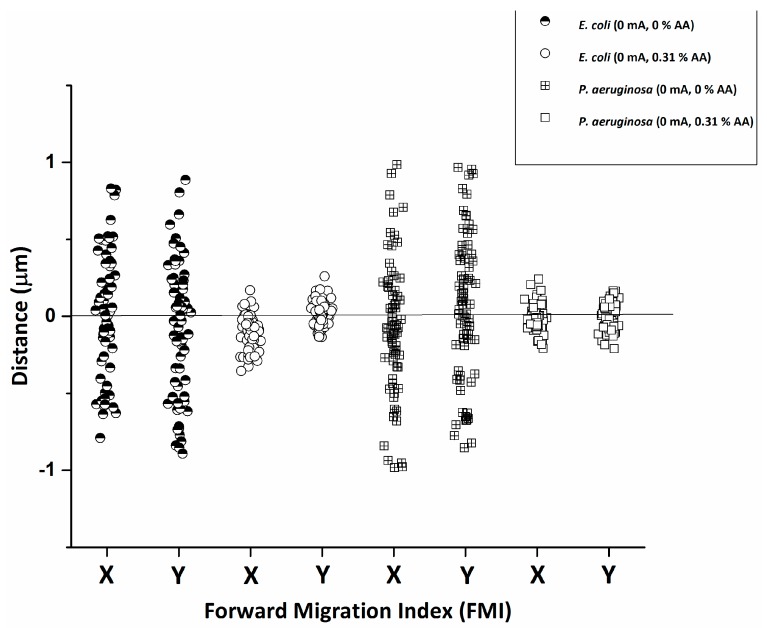
Vertical scatter indicating forward migration index (FMI) distribution in x and y directions for *E. coli* and *P. aeruginosa* comparing the effect of 0.31% AA alone on cells. Results indicate a drastic reduction in migration in both the x and y directions with the application of AA.

Both species experienced a reduction in average cellular speed (Figure 3), FMI (Figure 5), and directness (Table 1) with the introduction of 0.31% AA alone, relative to baseline (no AA). These results indicate that treatment with AA significantly impairs bacterial motility, even when used alone. Furthermore, treatment with AA has equivalent effects on both species, pointing to a broader, more conserved mechanism of action.

Studies have found that bacterial cellular Adenosine triphosphate (ATP) processes are disrupted by exposure to AA [19], which is one reason why AA has great potential as an antimicrobial agent. Weak acids can cross bacterial membranes more readily than strong acids, because of the equilibrium between their ionized and non-ionized forms, the latter of which can freely diffuse across hydrophobic membranes. This ultimately collapses the proton gradients necessary for ATP synthesis. When AA dissociates, it acidifies the cytoplasm, causing acid-induced proton unfolding and membrane/DNA damage [19]. This effect is specific to bacteria, because host somatic cells contain cholesterol, which controls cell permeability; the interior of the phospholipid bilayer is occupied by hydrophobic fatty acid chains such that the membrane is impermeable to water-soluble molecules and most biological molecules, including ions [28]. There are also other theories that support the notion of the reduction of bacterial motility from the application of electrical current. Direct electron transfer between the bacteria and the anode, which emits the electrical current, results in inactivation of the attached bacteria to surfaces [31]. A combination of electro-osmotic and electro-attractive forces may drive bacterial random motion during the interaction between the electric current and the bacterial cell [32].

The resulting average cellular speeds for *E. coli* in the presence of 0.31% AA were 0.091 ± 0.005, 0.085 ± 0.002, 0.072 ± 0.003, and 0.074 ± 0.002 µm/s for 0 mA, 0.07 mA, 0.125 mA, and 0.175 mA DC, respectively (Figure 3). The resulting average cellular speeds for *P*. *aeruginosa* in the presence of 0.31% AA were 0.085 ± 0.002, 0.075 ± 0.002, 0.081 ± 0.001, and 0.083 ± 0.002 µm/s for 0 mA, 0.07 mA, 0.125 mA, and 0.175 mA DC, respectively (Figure 3).

**Figure 6:**
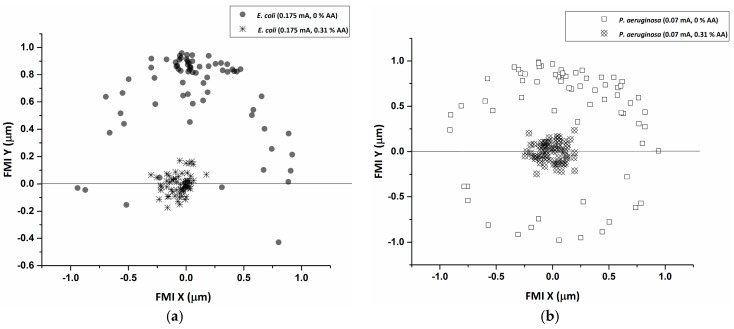
(**a**) The forward migration index (FMI) distribution for *E. coli* at the 0.175 mA setting in the presence and absence of 0.31% AA. The results indicate a drastic reduction in overall migration when 0.31% AA was applied in combination with an electrical current of 0.175 mA. (**b**) The FMI distribution for *P*. *aeruginosa* at a 0.07 mA setting in the presence and absence of 0.31% AA. The results indicate a drastic reduction in overall migration when 0.31% AA was applied in combination with an electrical current of 0.07 mA.

A reduction in cellular speed relative to baseline (0.31% AA, no current) (Figure 2 and Figure 3) (*p* < 0.05) was pronounced for both *E. coli* and *P*. *aeruginosa* upon electroceutical application. The most dramatic reductions in speed occurred at 0.070 mA DC for *P*. *aeruginosa*, while *E. coli* was equally impaired at higher currents (0.125 and 0.175 mA DC). These results suggest that there is an ideal current range in an electroceutical setting for *E. coli*, which would be upwards of 0.125 mA, while the ideal range for *P*. *aeruginosa* would be less than 0.125 mA for both single and multi-cue situations (namely 0.07 mA). However, a current of approximately 0.07 mA may be effective across a broader range of strains, meaning that a single application of current (with AA, especially) could provide powerful disinfection in the context of a wound infection. A very pronounced reduction in FMI was also observed at 0.175 mA DC for *E. coli* and at 0.07 mA for *P*. *aeruginosa* when single-cue ES was compared to our electroceutical approach (AA + current) (Figure 6a,b).

Clearly, *P*. *aeruginosa* responds well to both electrical stimulation and our electroceutical approach at 0.70 mA DC, while *E. coli* is more responsive at higher current (0.175 mA DC). It is not surprising that *E. coli* and *P*. *aeruginosa* respond differently to the electrical characteristics of their environment, as their chemotactic sensing systems differ. *P*. *aeruginosa* has a more complex sensing system than most microbes, with multiple chemotaxis genes that constitute several chemotaxis systems with defined functions [33]. *E. coli* uses a two-component regulatory system, consisting of an extracellular sensor and response regulator [30] that is more susceptible to oxidative stress than *P*. *aeruginosa* [33]. Hence, electroceutical device design should take into account the differing impacts that such disinfection strategies may have on several relevant wound pathogens. In the case of *P. aeruginosa*, quorum sensing (QS) plays a prominent role in its virulence and biofilm formation, which may offer an ideal target for anti-biofilm therapies.

Oxidative and nitrosative stresses, which can be augmented by electroceutical approaches, play important roles in bacterial inhibition/elimination in vivo. In particular, *E. coli* can be toxified by as little as 5 µM extracellular hydrogen peroxide [34,35], and because it is a small uncharged molecule, hydrogen peroxide diffuses across membranes rapidly and has the ability to cause profuse DNA damage when the intracellular concentration rises to 1 µM [24]. *P*. *aeruginosa*, on the other hand, has developed a multitude of defense mechanisms to tolerate stress conditions, even H_2_O_2_ at relatively high levels, but remains susceptible to other oxidative stressors [33]. The buildup of electrochemical oxidative products at the anode and cathode would occur with the application of electrical current, and while electrical stimulation alone may be a successful strategy for disinfection, in combination with AA, these effects are likely to be augmented. Combining AA with electrical stimulation should enhance disinfection, as AA has the ability to disrupt essential ATP processes and cause cellular DNA damage, thereby impairing both the ability to communicate and initiate defense mechanisms.

As motility is one microbial defense against opsonization by the host’s immune defenses, locomotive impairment would effectively trap bacterial cells, allowing for their clearance by migrating phagocytic leukocytes. Since migratory cell behavior in the presence of electrical cues is a naturally existing process within the human body, if we take the 0.1 µA/mm^2^ DC current density generated as a lower limit for directing leukocyte migration for wound healing, the corresponding current density settings tested here are likely to induce leukocyte migration that would accelerate the natural wound healing process while simultaneously inhibiting opportunistic pathogens. An applied electric field within the physiological range can also induce the directional electrotaxis of epithelial cells and fibroblasts, along with neutrophils and endothelial cells, suggesting a potential role in cellular positioning during wound healing [36]. Because phagocytic neutrophils and monocytes are up to 20 µm in diameter, have cell walls that actively prohibit the entry of water soluble molecules [28], and have a negative surface potential, unlike invading pathogens, they can migrate to the wound site unaffected. As a consequence, our electroceutical approach could create a bacterial trap that would accelerate the natural wound healing process, while augmenting bacterial clearance.

## 4. Conclusions

Using microfluidic platforms, optical microscopy, and bacterial tracking software, this study indicates that the combination of electrical stimulation and acetic acid could potentially be a viable electroceutical approach in controlling bacterial migration and preventing infection by pathogenic bacteria. Single-cue (current or AA only) and multi-cue (current and AA combined) experiments were conducted on multi-drug resistant strains of *E. coli* and *P. aeruginosa*. The cell motility of both *E. coli* and *P. aeruginosa* in 0.31% AA showed impairment at current values of 0.125 mA and 0.07 mA respectively, as well as a significant decrease in the FMI for both cell types. Electroceutical approaches to treating bacterial infection, such as those described in this experiment, could offer viable alternatives to overused antibacterial agents, and have potential applications in decreasing wound healing times.

## Figures and Tables

**Table 1:** Directness values for *E. coli* and *P. aeruginosa* affected by applied current and/or percentage of acetic acid (AA). Results indicate an increase in directness relative to baseline with the application of current alone, but a decrease in directness with the application of AA alone or with current.

Acetic Acid (AA) Concentration	Bacteria Type	0 mA	0.07 mA	0.125 mA	0.175 mA
0%	*E. coli*	0.74 ± 0.03	0.90 ± 0.02	0.82 ± 0.02	0.83 ± 0.02
	*P. aeruginosa*	0.79 ± 0.03	0.82 ± 0.02	0.87 ± 0.02	0.86 ± 0.05
0.31%	*E. coli*	0.16 ± 0.01	0.13 ± 0.01	0.10 ± 0.01	0.10 ± 0.01
	*P. aeruginosa*	0.11 ± 0.01	0.14 ± 0.01	0.12 ± 0.01	0.11 ± 0.01
